# *Porphyromonas gingivalis* Lipopolysaccharide Induced Proliferation and Activation of Natural Killer Cells in Vivo

**DOI:** 10.3390/molecules21081086

**Published:** 2016-08-19

**Authors:** Yuhua Wang, Wei Zhang, Li Xu, Jun-O Jin

**Affiliations:** 1 Department of Prosthodontics, Ninth People’s Hospital, College of Stomatology, Shanghai Jiao Tong University School of Medicine, Shanghai Key Laboratory of Stomatology, Shanghai 200011, China; heyaniy@hotmail.com; 2 Shanghai Public Health Clinical Center, Shanghai Medical College, Fudan University, Shanghai 201508, China; weiwei061215@126.com (W.Z.); wendengxvli@126.com (L.X.)

**Keywords:** *P. gingivalis* LPS, *E. coli* LPS, NK cells, dendritic cells

## Abstract

*Porphyromonas gingivalis* (*P. gingivalis*) lipopolysaccharide (LPS) promoted different innate immune activation than that promoted by *Escherichia coli* (*E. coli*) LPS. In this study, we examined the effect of *P. gingivalis* LPS on the proliferation and activation of natural killer (NK) cells in vivo and compared that function with that of *E. coli* LPS. Administration of *P. gingivalis* LPS to C57BL/6 mice induced stronger proliferation of NK cells in the spleen and submandibular lymph nodes (sLNs) and increased the number of circulating NK cells in blood compared to those treated with *E. coli* LPS. However, *P. gingivalis* LPS did not induce interferon-gamma (IFN-γ) production and CD69 expression in the spleen and sLN NK cells in vivo, and this was attributed to the minimal activation of the spleen and sLN dendritic cells (DCs), including low levels of co-stimulatory molecule expression and pro-inflammatory cytokine production. Furthermore, *P. gingivalis* LPS-treated NK cells showed less cytotoxic activity against Yac-1 target cells than *E. coli* LPS-treated NK cells. Hence, these data demonstrated that *P. gingivalis* LPS promoted limited activation of spleen and sLN NK cells in vivo, and this may play a role in the chronic inflammatory state observed in periodontal disease.

## 1. Introduction

*Porphyromonas gingivalis* (*P. gingivalis*) is a gram-negative anaerobic bacterium implicated as one of the major pathogens contributing to the development of chronic inflammatory periodontal diseases [1]. Lipopolysaccharide (LPS) in the surface components of *P. gingivalis* interacts with the innate immune cell-expressed toll-like receptors (TLRs), which provokes the release of chemokines and cytokines [2,3,4]. Activation of innate immune cells—such as neutrophils, natural killer (NK) cells, macrophages, and dendritic cells (DCs)—accelerates inflammatory responses, which contributes to the clearance of the invading pathogens [5]. Previous studies have shown that *P. gingivalis* LPS promotes a different innate immune activation compared to that of *Escherichia coli* (*E. coli*) LPS [3,6]. Systemic administration of *E. coli* LPS induces a potent stimulus for tumor necrosis factor-alpha (TNF-α) production in the mouse, whereas *P. gingivalis* LPS has a minimal stimulatory capacity on TNF-α production [7]. Moreover, *P. gingivalis* LPS activates macrophages weakly compared to *E. coli* LPS [8]. In addition, in vitro studies have shown that the capacity of *P. gingivalis* LPS to stimulate DCs is much weaker than that of *E. coli* LPS [9,10]. Furthermore, *P. gingivalis* LPS promotes production of interleukin-1β (IL-1β), TNF-α, and IL-6 in the human monocyte cell line THP-1 dependent on the TLR2 and c-Jun N-terminal kinase (JNK) pathway, while the cytokine induction by *E. coli* LPS was primarily via TLR4-NF-kappaB and TLR4-p38MAPK pathway [11,12]. *P. gingivalis* LPS promotes T helper 2 (Th2) immune responses, whereas *E. coli* LPS induces Th1 polarization [10]. Although previous studies have shown that *P. gingivalis* LPS plays a different and lower capacity in immune regulation compared to that of *E. coli* LPS, the activation of spleen NK cells in vivo by the *P. gingivalis* and *E. coli* LPSs has not been compared.

NK cells are one of the three major lymphocyte subsets that play important roles in the innate immune responses against bacterial and viral infection [13]. As the major interferon-gamma (IFN-γ) producers in the early stages, NK cells contribute to the promotion of inflammation in bacterial infection and sepsis [14,15]. Conversely, IFN-γ production can be triggered in NK cells as a result of contact with and recognition of target cells, which consequently promotes cytolysis of the target cells [15]. IFN-γ production in NK cells is mediated by interactions with other innate immune cells, such as macrophages and DCs, or pro-inflammatory cytokines, notably interleukin-12 (IL-12) and IL-2 [15,16]. In addition, in periodontitis lesions, infiltration and activation of NK cells have been demonstrated in inflamed gingival tissues and periodontal tissue destruction [17,18,19].

DCs promote enhancement of the NK cell functions including IFN-γ production and cytotoxic activities [3,16,20,21]. After exposure to microbial stimuli, DCs undergo a maturation process, which is defined as upregulation of co-stimulatory molecule expression and pro-inflammatory cytokine production [22,23]. IL-2 and IL-12, produced by stimulated DCs, play a fundamental role in the activation of NK cells [3,21,24]. *E. coli* LPS has been well characterized as potent inducers of IL-2 and IL-12 production in DCs [25], which induces NK cell activation in vivo [3,24,26]. Moreover, *P. gingivalis*-stimulated bone marrow-derived DCs (BMDCs) promote IFN-γ production in NK cells in vitro [27]. However, the function of *P. gingivalis* LPS in spleen and submandibular lymph node (sLN) DC activation and the interaction of DC and NK cells in vivo*,* have not been studied.

Because *P. gingivalis* LPS and *E. coli* LPS exhibited different effects in the activation of DC, macrophage, and T cells, we hypothesized that the effect of *P. gingivalis* LPS and *E. coli* LPS in NK cell activation may be different. The current study was undertaken to test this hypothesis.

## 2. Results

### 2.1. P. Gingivalis LPS Promotes Proliferation of Spleen and sLN NK Cells but Not Blood NK Cells in Vivo

**Figure 1:**
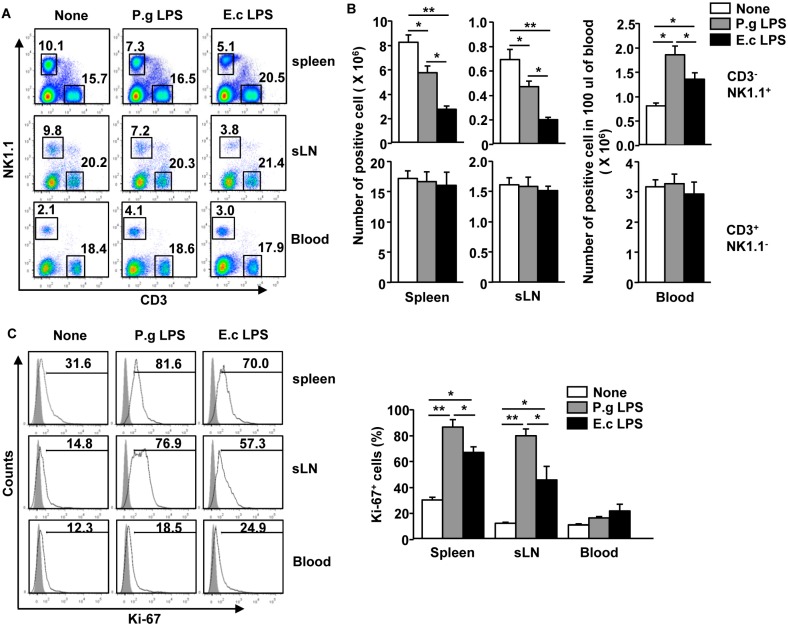
*P. gingivalis* LPS enhanced the proliferation of spleen and submandibular lymph node (sLN) natural killer (NK) cells in vivo. C57BL/6 mice were injected intravenously (*i.v.*) with 1 mg/kg of *P. gingivalis* (P.g) LPS or *E. coli* (E.c) LPS for 18 h. (**A**) The percentage of CD3^−^NK1.1^+^ and CD3^+^NK1.1^−^ cells in the spleen, sLNs, and blood were analyzed by flow cytometry; (**B**) Absolute cell number of CD3^−^NK1.1^+^ and CD3^+^NK1.1^−^ cells within live cells in the spleen, sLNs, and blood are shown; (**C**) Intranuclear expression levels of Ki-67 in the CD3^−^NK1.1^+^ cells in the spleen, sLNs, and blood are shown (**left panel**). The mean percentage of Ki-67 positive cells in the CD3^−^NK1.1^+^ in the spleen, sLN, and blood were illustrated (**right panel**). All data are representative or the average of analyses of six samples from three independent experiments. * *p* < 0.05, ** *p* < 0.01.

A previous study has shown that *E. coli* LPS promotes proliferation of NK cells in the spleen [20]. Therefore, we examined whether *P. gingivalis* LPS can also induce the proliferation of NK cells in vivo. C57BL/6 mice were treated intravenously (*i.v.*) with 1 mg/kg of *P. gingivalis* LPS or *E. coli* LPS for 18 h [28,29] Treatment with *P. gingivalis* LPS induced significant decreases in the number and frequency of CD3^−^NK1.1^+^ cells in the spleen and sLNs compared to null-treatment controls (Figure 1A,B), whereas the number of CD3^+^NK1.1^−^ cells were not changed by *P. gingivalis* LPS (Figure 1A,B). In contrast to the spleen and sLN NK cells, the number of blood NK cells were significantly increased by *P. gingivalis* LPS (Figure 1A lower panels and B right panels). Moreover, intranuclear staining of the Ki-67 antigen, a well-established measure of proliferative capacity, demonstrated that *P. gingivalis* LPS treatment substantially increased Ki-67 positive cells in the spleen and sLN CD3^−^NK1.1^+^ cells (Figure 1C), whereas blood CD3^−^NK1.1^+^ cells did not exhibit increased Ki-67 levels by *P. gingivalis* LPS (Figure 1C) *E. coli* LPS treatment led to a much stronger reduction in the cell number and frequency of CD3^−^NK1.1^+^ cells in the spleen and sLNs (Figure 1A,B), but the number of blood circulating NK cells and levels of Ki-67 levels in the spleen and sLNs were significantly lower than those induced by *P. gingivalis* LPS (Figure 1A–C). Thus, these data suggest that *P. gingivalis* LPS induced a stronger proliferating effect in the spleen and sLN NK cells compared to those cells treated with *E. coli* LPS.

### 2.2. P. Gingivalis LPS Did Not Upregulate IFN-γ Production and CD69 Expression in NK Cells

**Figure 2:**
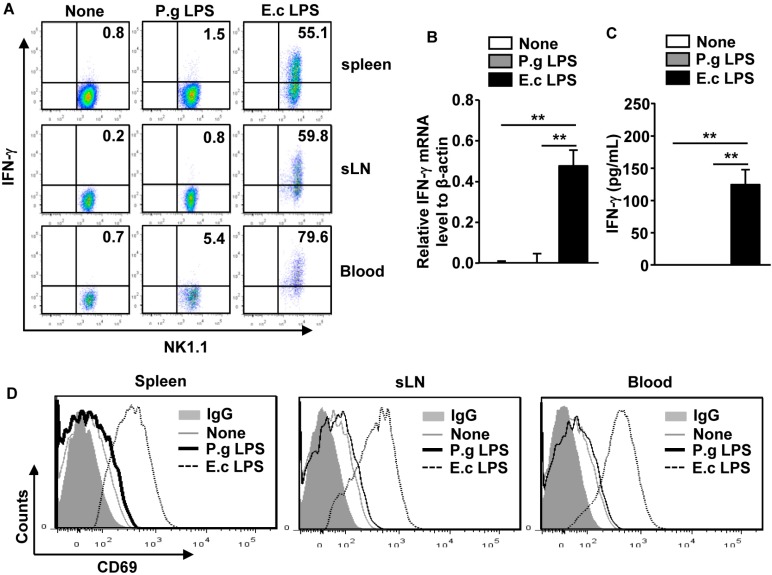
*P. gingivalis* LPS did not upregulate IFN-γ production and CD69 expression in the spleen and sLN NK cells. C57BL/6 mice were injected *i.v.* with 1 mg/kg of P.g LPS or E.c LPS. Six hours after injection, splenocytes were further cultured with monensin solution for 4 h. (**A**) Intracellular IFN-γ production levels in the CD3^−^NK1.1^+^ cells in the spleen, sLNs, and blood were analyzed by flow cytometry; (**B**) C57BL6 mice were treated with the LPS for 6 h; subsequently NK cells were isolated from the spleen. The mRNA levels of IFN-γ were measured in the spleen NK cells; (**C**) Isolated NK cells were further incubated in the culture medium for 4 h. The IFN-γ concentration in the cultured medium was measured by ELISA; (**D**) The CD69 expression level in the spleen, sLN, and blood NK cells were analyzed 6 h after LPS treatment. All data are representative or the average of analyses of six samples from three independent experiments. ** *p <* 0.01.

Activated NK cells produce high levels of IFN-γ, and *E. coli* LPS has been well defined as a strong inducers of IFN-γ in NK cells [14,15]. We examined whether *P. gingivalis* LPS also promotes the production of IFN-γ in the spleen, sLNs, and blood NK cells in vivo. C57BL/6 mice were injected *i.v.* with 1 mg/kg of either *P. gingivalis* LPS or *E. coli* LPS. Six h after the injection, splenocytes were further incubated with a monensin solution for 4 h. Consistent with previous reports, *E. coli* LPS promoted marked increases in the IFN-γ-producing NK cells in the spleen, sLNs, and blood (Figure 2A), whereas *P. gingivalis* LPS did not induce this increase in the spleen, sLNs, and blood (Figure 2A). To further define IFN-γ production in NK cells, C57BL/6 mice were injected *i.v.* with 1 mg/kg of either *P. gingivalis* LPS or *E. coli* LPS. Six h after the injection, NK cells were isolated from the spleen and mRNA levels and production levels of IFN-γ were measured. The mRNA level of IFN-γ in the spleen NK cells were not increased by the *P. gingivalis* LPS treatment compared to the null-treatment control (Figure 2B), whereas the *E. coli* LPS treatment exhibited significant increases in the IFN-γ mRNA levels (Figure 2B) Consistent with the mRNA levels, the production levels of IFN-γ in the cultured medium of the isolated NK cells were not increased by the *P. gingivalis* LPS treatment, whereas *E. coli* LPS treated spleen NK cells produced high levels of IFN-γ (Figure 2C). In addition, the *E. coli* LPS treated mice, but not the *P. gingivalis* LPS treated mice, displayed an increased level of the CD69 expression, an early NK cell activation marker [30], on the surface of spleen, sLN, and blood NK cells compared to the null-treatment control (Figure 2D). Thus, these data suggest that *P. gingivalis* LPS is not able to induce full activation of spleen, sLN, and blood NK cells in vivo.

### 2.3. P. Gingivalis LPS Induced Minimal Activation of Spleen DCs

**Figure 3:**
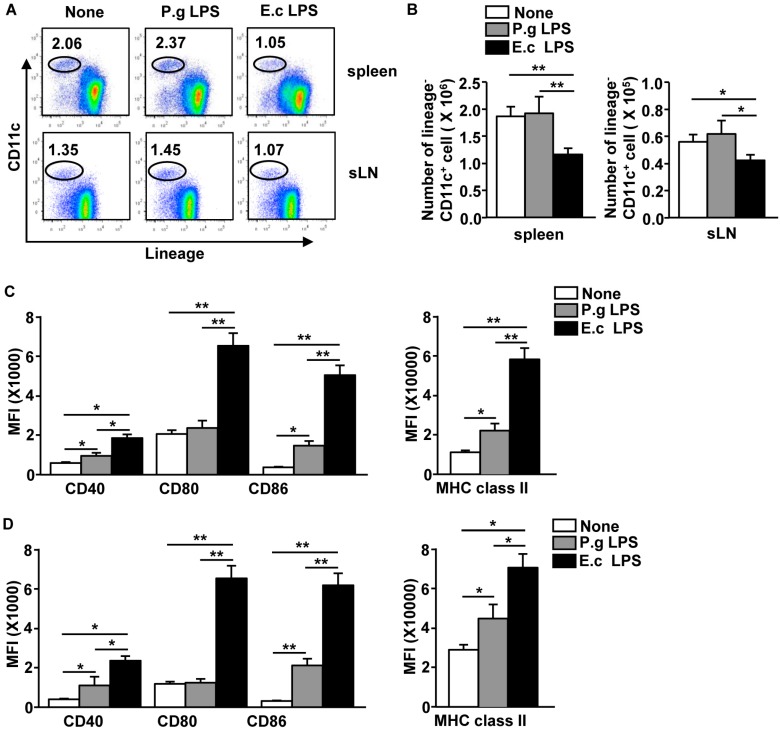
*P. gingivalis* LPS weakly activated spleen and sLN DCs. C57BL/6 mice were injected *i.v.* with 1 mg/kg of P.g LPS or E.c LPS for 12 h. (**A**) The percentage of lineage^−^CD11c^+^ DCs in the spleen (upper panel) and sLN (lower panel) was analyzed by flow cytometry; (**B**) Absolute numbers of live, lineage^−^CD11c^+^ cells in the spleen and sLN were shown; (**C**,**D**) Mean fluorescence intensity (MFI) of indicated surface molecules in the spleen (**C**) and sLN (**D**) was analyzed on a flow cytometry. All data are representative of or the average of analyses of six independent samples (two mice per experiment, total three independent experiments). * p < 0.05. ** p < 0.01.

Our data indicated that *P. gingivalis* LPS did not fully induce spleen, sLN, and blood NK cell activation in vivo. This conclusion prompted us to examine in vivo DC activation by *P. gingivalis* LPS, because mature DCs interacted and induced NK cell activation. C57BL/6 mice were injected *i.v.* with 1 mg/kg of *P. gingivalis* LPS or *E coli* LPS for 12 h. The *P. gingivalis* LPS injection did not alter the population, frequency, or number of spleen and sLN DCs compared to the null-treatment mice (Figure 3A,B). However, treatment with *E. coli* LPS led to substantial decreases in the frequency and number of spleen and sLN DCs (Figure 3A,B). The expression levels of co-stimulatory molecules and MHC class II were considerably increased by the *P. gingivalis* LPS treatment in the spleen (Figure 3C) and sLN DCs (Figure 3D) compared to the null-treatment control mice. The levels of those molecules were significantly lower than the *E. coli* LPS-induced upregulation (Figure 3C,D). Thus, these data suggest that the effect of *P. gingivalis* LPS in the spleen and sLN DC activation in vivo was much weaker than those induced by *E. coli* LPS.

### 2.4. P. Gingivalis LPS Failed to Induce IL-2, IL-12 and IL-18 Production in Spleen DCs

**Figure 4:**
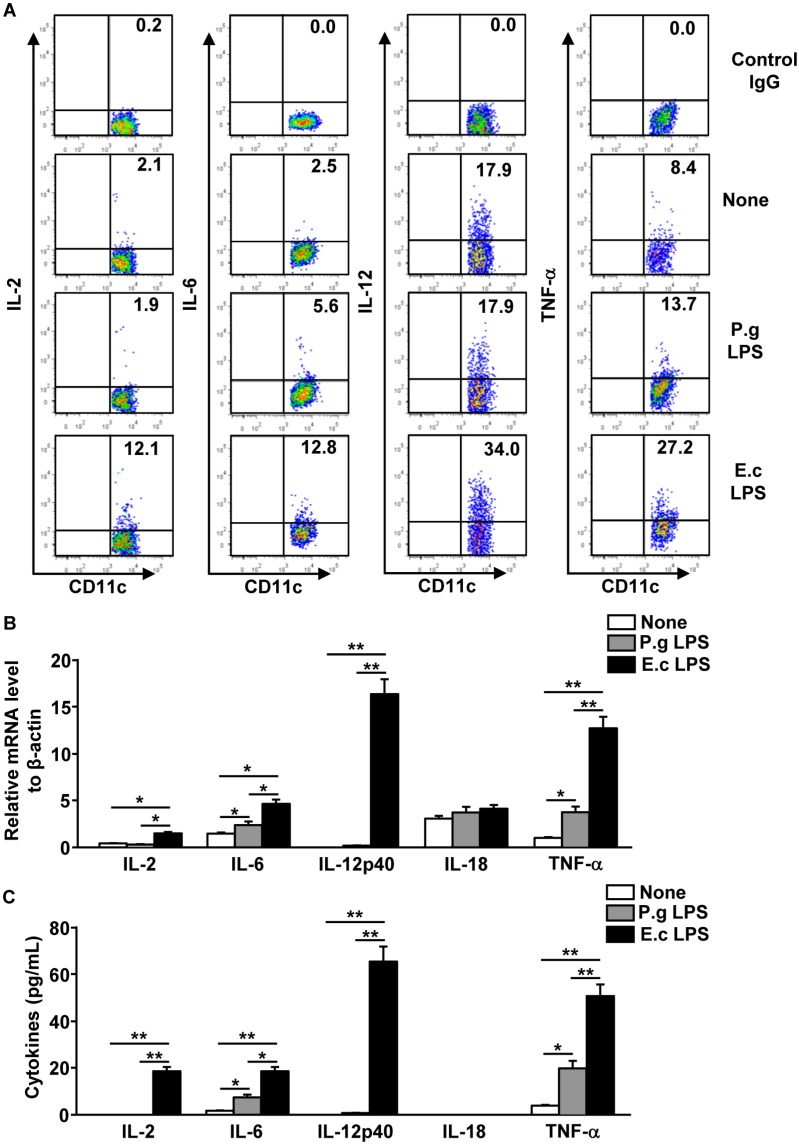
*P. gingivalis* LPS weakly induced pro-inflammatory cytokine production in spleen DCs. C57BL/6 mice were injected *i.v.* with 1 mg/kg of P.g LPS or E.c LPS. (**A**) Four hours after injection, the spleen was harvested from the mice and 1 × 10^6^ splenocytes were further incubated with monensin for an additional 2 h. Levels of intracellular cytokine production in the spleen DCs were analyzed by flow cytometry; (**B**) CD11c^+^ spleen DCs were isolated 6 h after injection, and measured to mRNA levels of pro-inflammatory cytokines were measured; (**C**) The isolated spleen DCs (0.1 × 10^6^) were cultured in 1 mL of RPMI-1640 for 4 h in vitro. The concentrations of indicated cytokines were measured in the cultured medium by ELISA. All data are representative of or the average of analyses of six individual mice in each group (two mice per experiment, total three independent experiments). * *p <* 0.05, ** *p* < 0.01.

In the literature, activated DCs have been reported to produce IL-2, IL-12, and IL-18, which induce IFN-γ production in NK cells [31,32,33]. We therefore assessed whether *P. gingivalis* LPS can induce the production of those cytokines in the spleen DCs, because the in vivo treatment of *P. gingivalis* LPS did not induce the production of IFN-γ in NK cells. C57BL/6 mice were injected *i.v.* with *P. gingivalis* LPS or *E. coli* LPS. Four hours after injection, splenocytes were further incubated with monensin for an additional 2 h and the intracellular IL-2, IL-6, IL-12p40, and TNF-α levels were measured in the spleen DCs. The *E. coli* LPS treatment in the mice led to a dramatic increase in the IL-2, IL-6, IL-12p40, and TNF-α-producing DCs in the spleen (Figure 4A). Unlike the *E. coli* LPS treatment, which substantially induced the production of all these cytokines, the *P. gingivalis* LPS treatment did not affect the production of the IL-2 or IL-12p40 in spleen DCs (Figure 4A). IL-6 and TNF-α levels were considerably increased by *P. gingivalis* LPS in the spleen DCs, although those levels were much lower than induced by *E. coli* LPS induced (Figure 4A).

Next, spleen DCs were purified by microbead-conjugated anti-CD11c antibodies (Milteni Biotec, Bergisch Gladbach, Germany) after 4 h of LPS stimulation. The isolated spleen DCs were measured for the mRNA levels of those cytokines and *P. gingivalis* LPS were determined not to induce increases in the IL-2, IL-12p40, and IL-18 mRNA levels in the spleen DCs compared to the null-treatment control DCs (Figure 4B), whereas the IL-6 and TNF-α mRNA levels were significantly increased by *P. gingivalis* LPS (Figure 4B). *E. coli* LPS-treated spleen DCs exhibited much higher mRNA levels of those cytokines than the null-treatment or *P. gingivalis* LPS-treated spleen DCs (Figure 4B). We also examined the production levels of those cytokines in the spleen DCs. Spleen DCs were isolated after 6 h of LPS treatment and were cultured in vitro for an additional 4 h. The cytokine concentration in the cultured medium was then measured. Consistent with mRNA levels, the concentration of IL-2, IL-12p40, and IL-18 in the cultured medium of *P. gingivalis*-treated spleen DCs did not increase (Figure 4C), whereas the *E. coli* LPS-treated spleen DCs substantially increased the concentration of cytokines in the medium compared to the null-treatment control DCs (Figure 4C). Meanwhile, the IL-6 and TNF-α concentrations in the *P. gingivalis* LPS-stimulated spleen DCs were considerably increased in comparison to the null-treatment controls. Thus, these data suggest that *P. gingivalis* LPS did not induce IL-2, IL-12, and IL-18 production in spleen DCs.

### 2.5. P. Gingivalis LPS Induced Minimal Levels of Cytotoxic Activity in Spleen NK Cells

**Figure 5:**
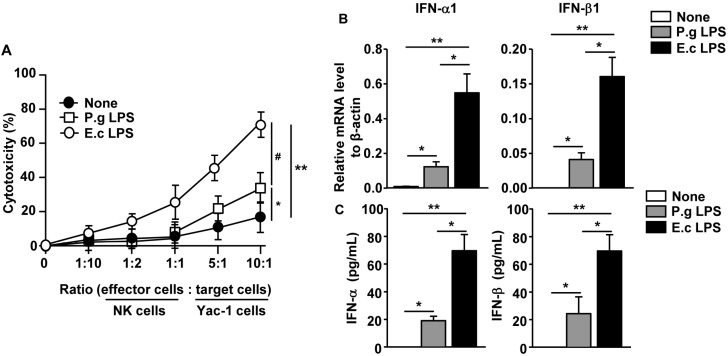
*P. gingivalis* LPS promoted a weak level of cytotoxic activity in the spleen NK cells. C57BL/6 mice were injected *i.v.* with 1 mg/kg of P.g LPS or E.c LPS and 24 h later, were injected again with the same amount of LPS. (**A**) Twenty-four hours after last treatment, NK cells were isolated from the spleen and co-cultured with Yac-1 cell by the indicated ratio for 6 h. Cytotoxicity was measured by the lactate dehydrogenase (LDH) assay. # *p < 0.05* *P. gingivalis* LPS versus *E. coli* LPS group, * *p* < 0.05 None versus *P. gingivalis* LPS group, ** *p* < 0.01 None versus *E. coli* LPS group; (**B**) mRNA levels of IFN-α1 and IFN-β1 in splenocyte after 12 h of LPS treatment were measured; (**C**) The concentration of IFN-α and IFN-β in the serum was measured by ELISA. All data are representative of or the average of analyses of six individual mice in each group (two mice per experiment, total three independent experiments). * *p* < 0.05, ** *p* < 0.01.

The cytotoxic activities of spleen NK cells by *P. gingivalis* and *E. coli* LPS were examined. C57BL/6 mice were injected *i.v.* with 1 mg/kg *P. gingivalis* and *E. coli* LPS and 24 h later, they were injected again with the same amount of LPS. Twenty-four hours after the last injection, NK cells (effector cells) were isolated from the spleen and co-cultured with Yac-1 cells (target cells) for detection of MHC-negative target cell killing. The presence of *P. gingivalis* LPS-stimulated spleen NK cells led to significantly increased levels of cytotoxic activity compared to the null-treatment spleen NK cells (Figure 5A), whereas *E. coli* LPS-treated spleen NK cells exhibited greater cytolysis of Yac-1 cells than *P. gingivalis* LPS-induced cells (Figure 5A).

Because the cytotoxic activities of NK cells are dependent on type I IFNs [24], we next measured the levels of type I IFNs in the LPS-treated mice. The *P. gingivalis* LPS treatment promoted considerable increases in the mRNA levels of type I IFNs in the spleen and protein levels of type I IFNs in serum compared to the null-treatment controls (Figure 5B,C). In contrast, the *E. coli* LPS treatment substantially elevated the mRNA and protein levels of type I IFNs, and the levels were greater than that of the *P. gingivalis* LPS-induced levels (Figure 5B,C). Hence, these data demonstrated that *P. gingivalis* LPS promotes minimal levels of cytotoxic activity in spleen NK cells, attributed to low levels of type I IFN production.

## 3. Discussion

In this study, we demonstrated that *P. gingivalis* LPS and *E. coli* LPS elicited different effects on NK cell proliferation and activation in vivo. *E. coli* LPS induced activation of NK cells including IFN-γ production, CD69 expression, and cytotoxic activity, while *P. gingivalis* LPS induced minimal activation of NK cells. In contrast to the activation effect, *P. gingivalis* LPS promoted a greater proliferation in the spleen and sLN and increased the number of NK cells circulating in blood relative to *E. coli* LPS. The different effects of these two LPSs are consistent with several previous reports, which have investigated the macrophages and T cells [6,7,8]. Hence, the minimal activation effect of *P. gingivalis* LPS in the macrophages, T cells, and NK cells may be a potential reason for *P. gingivalis*-induced chronic inflammatory diseases. Further investigation will address this possibility and elucidate the specific role of the immune cells in *P. gingivalis*-induced chronic periodontitis.

We also determined that *P. gingivalis* LPS did not fully activate spleen and sLN DCs as indicated by the low levels of co-stimulatory molecule expression and pro-inflammatory cytokine production, while *E. coli* LPS promoted strong activation of those cells in vivo. Although previous studies demonstrated that *P. gingivalis* LPS induced DC activation in vivo [10], the activation efficiencies of *E. coli* and *P. gingivalis* LPSs were not compared. Moreover, a recent in vitro study also demonstrated that *P. gingivalis* LPS induced lower secretion levels of IL-12 and IFN-γ in BMDCs than those induced by *E. coli* LPS [9]. In addition, we also found that *P. gingivalis* LPS did not induce the changes in the spleen and sLN DC number, whereas the *E. coli* LPS treatment induced a markedly decreased number of spleen and sLN DCs. Because fully matured DCs undergo exhaustion, anergy, or apoptosis following T-cell activation [34,35,36], *E. coli* LPS might induce full activation but *P. gingivalis* LPS might not induce full activation of spleen DCs. Therefore, our results provide several lines of evidence that *P. gingivalis* LPS does not fully activate spleen and sLN DCs in vivo, and clearly demonstrate the unexplored in vivo function of *P. gingivalis* LPS in the spleen and sLN DCs.

Activated and matured DCs promote IFN-γ production in NK cells [21,24,26]. Consistent with previous reports, *E. coli* LPS induced DC activation and IFN-γ production in NK cells [21,24]. Moreover, activated DCs produced IL-2 and IL-12, which are crucial promoters of the production of IFN-γ in the NK cells [21,24,26]. In this study, we also found that *E. coli* LPS promotes IL-2 and IL-12 production in the spleen DCs in vivo. In contrast, *P. gingivalis* LPS did not induce IL-2 and IL-12 secretion in the spleen DCs although the DCs showed increased expression of co-stimulatory molecules. The data suggest that these cytokines may be essential for IFN-γ production in the NK cells in vivo, and upregulation of co-stimulatory molecule expression in DCs may not be required for IFN-γ production in NK cells. Our further investigation will determine the function of co-stimulatory molecules in the spleen DCs on the NK cells by LPS treatment using IL-2-, IL-12-, and IL-18-depleted mice, or a blockade antibody.

NK cells have the ability to kill MHC-negative targets [24,37]. Previous studies have demonstrated that the NK cell-mediated cytotoxic effect is dependent on type I IFNs [24,38,39]. We also found that *P. gingivalis* LPS induced upregulation of type I IFNs, and consequently promotes cytotoxic activities in the spleen NK cells against Yak-1 target cells. Type I IFNs can be produced by many immune cell types including NK cells, B cells, T cells, macrophages, and DCs [40,41]. Plasmacytoid DCs (pDCs) have been identified as a potent producer of type I IFNs in response to viral antigens; however, pDCs do not produce type I IFNs in the spleen in vivo in response to *E. coli* LPS [42]. Unlike the in vivo study, in vitro generated BMDCs produce type I IFNs after *E. coli* LPS stimulation [24]. Moreover, human and mouse macrophages derived in vitro from precursors can also produce type I IFNs by *E. coli* LPS [43,44]. Therefore, the precise contribution of type I IFN-producing immune cells in the response to *E. coli* or *P. gingivalis* LPS in the circumstances of in vivo still remains to be studied. Future studies will define the cellular and molecular mechanisms of type I IFN production in the immune cells by *E. coli* or *P. gingivalis* LPS stimulation in the mouse model in vivo.

NK cells are abundant in periodontitis lesions, and activation of NK cells has been linked to inflammation of periodontal tissue [19]. Infiltrated NK cells in inflamed gingival tissue have been observed [19]. Moreover, it has been shown that NK cells are increased in chronic periodontitis [17,18,19]. However, the activation of NK cells was not fully investigated in chronic periodontitis. Because *P. gingivalis* LPS did not induce full activation of NK cells in sLNs but promoted proliferation of NK cells in vivo, those inactivated NK cells may be not able to be clear the *P. gingivalis* bacterium and this may contribute to the persistence of *P. gingivalis* and development of chronic periodontitis. Further studies will define the state of NK cells in chronic periodontitis.

In summary, we found that *P. gingivalis* LPS induces proliferation of NK cells in the spleen and sLNs, but those NK cells did not produce IFN-γ nor expressed CD69. Moreover, *P. gingivalis* LPS treated NK cells promote low levels of cytotoxic activates compared to *E. coli* LPS. Those low levels of NK cell activation in response to *P. gingivalis* LPS was attributed to the minimal activation of DCs and type I IFN production.

## 4. Materials and Methods

### 4.1. Animals

C57BL/6 mice were purchased from the Shanghai Public Health Clinical Center, and kept under pathogen-free conditions. The mice were maintained in a room with controlled temperature (20–22 °C), humidity (50%–60%), and light (12 h:12 h), and free access to standard rodent chow and water. This study was performed in strict accordance with the recommendations in the Guide for the Care and Use of Laboratory Animals of the Shanghai Public Health Clinical Center. The protocol was approved by the Committee on the Ethics of Animal Experiments of the Fudan University (Permit Number: SYXK-2010-0098). Mice were sacrificed by CO_2_ inhalation euthanasia, and all efforts were made to minimize suffering.

### 4.2. Antibodies and Reagents

The cells were stained and analyzed on a FACSAria II (Becton Dickinson, San Diego, CA, USA), with dead cells excluded by DAPI staining. The following fluorescence-conjugated Abs were used: CD3 (17A2), NK1.1 (PK136), Ki-67 (16A8), CD69 (H1.2F3), CD11c (N418), CD40 (HM40-3), CD80 (16-10A1), CD86 (GL-1), MHC class II (M5/114.15.2), anti-IFN-γ (XMG1.2), IL-2 (JES6-5H4), IL-6 (MP5-20F3), IL-12p40 (C15.6), and TNF-α (MP6-XT22) and obtained from BioLegend (San Diego, CA, USA). The *P. gingivalis* LPS (LPS-PG; standard) and *E. coli* LPS (LPS-EK; ultrapure) used in this study were purchased from InvivoGen (San Diego, CA, USA).

### 4.3. NK Cell Analysis

The spleens were cut into small fragments and digested with 2% fetal bovine serum (FBS) containing collagenase for 20 min at room temperature. Cells from the digest were centrifuged to a pellet, and the pellet was re-suspended in 5 mL of a 1.077 histopaque (Sigma-Aldrich, St. Louis, MO, USA). Additional histopaque was layered below and EDTA-FBS was layered above the cell suspension, which was then centrifuged at 1700 *g* for 10 min. For blood NK cell analysis, the blood cells were layered above the 1.077 histopaque, and then centrifuged at 1700 *g* for 10 min. The light density fraction (<1.077 g/cm^3^) was collected and stained with fluorescence-labeled CD3 and NK1.1 monoclonal antibodies. The sLNs were placed in a grinder and processed with a tissue homogenizer. Tissue homogenates were filtered through a 100 μm nylon mesh, washed, and the erythrocytes were removed with an Ammonium-Chloride-Potassium (ACK) lysing buffer (Thermo Fisher Scientific, Waltham, MA, USA). The single cells were resuspended in culture medium and stained with fluorescence-labeled CD3 and NK1.1 monoclonal antibodies.

### 4.4. DC Analysis

Spleen and sLN DCs were analyzed as described elsewhere [23,45,46]. After density cut by 1.077 histopaque from digested spleen as shown in NK cell analysis, the light density fraction (<1.077 g/cm^3^) was collected and incubated for 30 min with the following FITC-conjugated monoclonal antibodies (mAbs): anti-CD3 (17A2), anti-Thy1.1 (OX-7), anti-B220 (RA3-6B2), anti-Gr1 (RB68C5), anti-CD49b (DX5), and anti-TER-119 (TER-119) (BioLegend, San Diego, CA, USA). The lineage^−^CD11c^+^ cells were defined as DCs. Analysis was carried out on a FACS Aria II (Becton Dickinson, San Diego, CA, USA).

### 4.5. Cytotoxicity Assay

Activated spleen NK cells were isolated and co-cultured with Yac-1 cells from a mouse lymphoma cell line in V-bottomed 96-well plates in 200 μL of RPMI 1640 10% fetal calf serum for 24 h. 50,000 target Yac-1 cells were co-cultured in triplicate with 5000 (1:10), 25,000 (1:2), 50,000 (1:1), 250,000 (5:1), and 500,000 (10:1) NK cells, respectively. Cytotoxicity was determined by the lactate dehydrogenase (LDH) assay. The LDH assay was performed using a LDH Cytotoxicity Assay Kit (Roche, Basel, Switzerland) according to the manufacturer’s instructions.

### 4.6. Ex Vivo Cell Stimulation and Intracellular Cytokine Staining

As described in detail previously [45,47], single cell suspensions prepared from spleen were incubated in vitro for 4 h with monensin solution (Biolegend). For intracellular cytokine staining, cells were stained for surface molecules first, then fixed and permeabilized with Cytofix/Cytoperm buffer (eBioscience, San Diego, CA, USA) and subsequently incubated with anti-cytokine antibodies in Perm/Wash buffer (eBioscience) for 30 min. Control staining with isotype control IgGs was performed in all experiments.

### 4.7. Real-Time qPCR

Total RNA was reverse-transcribed into cDNA using Oligo (dT) and M-MLV reverse transcriptase (Promega, Madison, WI, USA). The cDNA was subjected to real-time PCR amplification (Qiagen, Hilden, Germany) for 40 cycles with annealing and extension temperature at 60 °C, on a LightCycler 480 Real-Time PCR System (Roche, Basel, Switzerland). Primer sequences are: mouse β-actin forward, 5′-TGGATGACGATATCGCTGCG-3′; reverse, 5′-AGGGTCAGGATACCTCTCTT-3′, IFN-α1 forward, 5′-ACCTCAGGAACAAGAGAGCC-3′; reverse, 5′-CTGCGGGAATCCAAAGTCCT-3′, IFN-β1 forward, 5′-TAAGCAGCTCCAGCTCCAAG-3′; reverse, 5′-CCCTGTAGGTGAGGTTGATC-3′,IL-2 forward, 5′-ATGAACTTGGACCTCTGCG-3′; reverse, 5′-GGGCTTGTTGAGATGATGC-3′, IL-6 forward, 5′-AACGATGATGCACTTGCAGA-3′; reverse, 5′-GAGCATTGGAAATTGGGGTA-3′, IL-12p40 forward, 5′-CACATCTGCTGCTCCACAAG-3′; reverse, 5′-CCGTCCGGAGTAATTTGGTG-3′, IL-18 forward, 5′-AGGACACTTTCTTGCTTGCC-3′; reverse, 5′-CCTCGGGTATTCTGTTATGGA-3′, TNF-α forward, 5′-CCTTTCACTCACTGGCCCAA-3′; reverse, 5′-AGTGCCTCTTCTGCCAGTTC-3′.

### 4.8. ELISA Assay

The IL-2, IL-6, IL-12p40, and TNF-α ELISA kits were purchased from Biolegend. The IFN-α and IFN-β ELISA kits were purchased from eBioscience. The concentration of proteins in the cultured medium and serum were measured in triplicate using standard ELISA kits, with standard cytokine preparations being used as the internal controls.

### 4.9. Statistical Analysis

Results were expressed as the mean ± standard error of the mean (SEM). Data sets were analyzed by one-way ANOVA using the Tukey multiple comparison test with GraphPad Prism 4. *p* values smaller than 0.05 were considered to be statistically significant.

## 5. Conclusions

In conclusion, the present study demonstrates that *P. gingivalis* LPS induced low levels of activation and high levels of proliferation in the spleen and sLN NK cells in vivo. The low levels of activation and increased number of NK cells observed may lead to the persistence and survival of *P. gingivalis*, resulting in *P. gingivalis*-induced chronic periodontal diseases.
